# Modulation of Human Mesenchymal Stem Cell Immunogenicity through Forced Expression of Human Cytomegalovirus US Proteins

**DOI:** 10.1371/journal.pone.0036163

**Published:** 2012-05-30

**Authors:** Melisa A. Soland, Mariana G. Bego, Evan Colletti, Christopher D. Porada, Esmail D. Zanjani, Stephen St. Jeor, Graça Almeida-Porada

**Affiliations:** 1 Department of Animal Biotechnology, University of Nevada, Reno, Nevada, United States of America; 2 Laboratory of Human Retrovirology, Institut de Recherches Cliniques de Montréal (IRCM), Montréal, Québec, Canada; 3 Department of Microbiology and Immunology, School of Medicine, University of Nevada, Reno, Nevada, United States of America; Instituto de Engenharia Biomédica, University of Porto, Portugal

## Abstract

**Background:**

Mesenchymal stem cells (MSC) are promising candidates for cell therapy, as they migrate to areas of injury, differentiate into a broad range of specialized cells, and have immunomodulatory properties. However, MSC are not invisible to the recipient's immune system, and upon in vivo administration, allogeneic MSC are able to trigger immune responses, resulting in rejection of the transplanted cells, precluding their full therapeutic potential. Human cytomegalovirus (HCMV) has developed several strategies to evade cytotoxic T lymphocyte (CTL) and Natural Killer (NK) cell recognition. Our goal is to exploit HCMV immunological evasion strategies to reduce MSC immunogenicity.

**Methodology/Principal Findings:**

We genetically engineered human MSC to express HCMV proteins known to downregulate HLA-I expression, and investigated whether modified MSC were protected from CTL and NK attack. Flow cytometric analysis showed that amongst the US proteins tested, US6 and US11 efficiently reduced MSC HLA-I expression, and mixed lymphocyte reaction demonstrated a corresponding decrease in human and sheep mononuclear cell proliferation. NK killing assays showed that the decrease in HLA-I expression did not result in increased NK cytotoxicity, and that at certain NK∶MSC ratios, US11 conferred protection from NK cytotoxic effects. Transplantation of MSC-US6 or MSC-US11 into pre-immune fetal sheep resulted in increased liver engraftment when compared to control MSC, as demonstrated by qPCR and immunofluorescence analyses.

**Conclusions and Significance:**

These data demonstrate that engineering MSC to express US6 and US11 can be used as a means of decreasing recognition of MSC by the immune system, allowing higher levels of engraftment in an allogeneic transplantation setting. Since one of the major factors responsible for the failure of allogeneic-donor MSC to engraft is the mismatch of HLA-I molecules between the donor and the recipient, MSC-US6 and MSC-US11 could constitute an off-the-shelf product to overcome donor-recipient HLA-I mismatch.

## Introduction

Mesenchymal stem cells (MSC) are promising candidates for use in cellular replacement therapies, since they have the inherent ability to migrate to areas of inflammation and injury and participate in tissue repair [1], [2], [3], [4]. This beneficial effect is due to MSC's ability to differentiate into several different cell types, to release soluble factors that inhibit apoptosis and promote healing, and to stimulate and/or support resident stem/progenitor cells [5], [6], [7], [8]. Another advantage of MSC over other putative stem cells is that MSC can be harvested using straightforward procedures, and expanded in vitro to obtain large numbers of cells, without losing their original potential. Besides their proliferation and differentiation potentials, MSC have also shown immunomodulatory capabilities at multiple levels. In vitro studies demonstrated that MSC express intermediate levels of HLA class I and lack expression of HLA class II and other co-stimulatory molecules, resulting in immune evasion during allogeneic transplantation [9]. Moreover, MSC were shown to inhibit proliferation of natural killer (NK) cells, T and B lymphocytes, and impair dendritic cell maturation during in vitro assays [10], [11], [12], [13], [14], [15]. In vivo, when systemically administered, MSC have been shown to extend survival of skin allografts in baboons [16], and are able to ameliorate graft-versus-host disease (GVHD) in human patients [17].

However, other studies in murine and swine models have provided evidence that MSC are not invisible to the recipient's immune system, and that upon in vivo administration, MSC are able to trigger immune responses, resulting in rejection of the transplanted cells [18], [19], [20], [21], [22]. These studies showed that one of the major factors responsible for the failure of donor MSC to engraft is the mismatch of HLA class I molecules between the donor and the recipient [18], [19], [23]. Furthermore, using a rhesus macaque model, Isakova et al. demonstrated that allogeneic MSC, upon intracranial injection, were able to induce an immune response that was dependent on cell dose and the degree of donor-host HLA-I mismatch. This study also showed that the immune response was mediated by NK and cytotoxic T cells [24]. Therefore, for those instances in which transplant timing or underlying disease preclude the use of autologous MSC, it is imperative to find ways to overcome donor-recipient HLA-I mismatch, so that the full therapeutic benefit from allogeneic MSC transplants can be achieved. One possible approach would be to engineer MSC in such a way that the expression of HLA-I molecules would be decreased or absent. This would allow the generation of an “off-the-shelf” universal donor MSC that could serve as an immediate source of cells to anyone in need, and would also thwart high costs of a personalized MSC therapy.

Human cytomegalovirus (HCMV), a virus ubiquitously present in humans, has developed several strategies to evade cytotoxic T lymphocyte (CTL) and NK cell recognition [25], [26], [27], [28]. HCMV avoids CTL attack by producing proteins, coded for by the unique short (US) region of the genome, that downregulate HLA-I surface expression by different mechanisms. US2 and US11 proteins induce translocation of HLA class I heavy chains from the endoplasmic reticulum (ER) to the cytosol, where they are degraded by the proteasome. US3 protein retains HLA class I molecules inside the ER, while US6 protein prevents peptide translocation and loading onto the HLA class I molecules [29], [30], [31].

In this study, we genetically engineered MSC to express each of these US proteins, in order to determine which one would be able to better downregulate HLA-I expression, and thus shield MSC from CTL attack. It is important to note that failure to engage class I inhibitory receptors on the NK cells by putative target cells can lead to increased susceptibility of these cells to NK cell lysing. Therefore, we also investigated whether preventing MSC HLA-I expression by US proteins would lead to enhanced NK killing of the MSC. Here we report that MSC engineered to express US6 (MSC-US6) or US11 (MSC-US11) exhibited the most pronounced reduction in HLA-I surface levels, and decrease in human and sheep lymphocyte proliferation, while NK killing assays showed that MSC-US11 were protected from NK cytotoxic effects. Using the fetal sheep as a model of human stem cell transplantation, we have previously reported on the ability of human-derived MSC to engraft and generate hepatocytes [5], [32]. In this model, both CTL and NK-like cells are present in circulation at the time of transplant, but the immune system is still immature, allowing for the engraftment of human cells without additional manipulation [33]. In the present studies, we used human hepatocyte formation in fetal sheep as a paradigm to investigate whether the expression of US6 or US11 on MSC would allow higher levels of engraftment, and differentiation into liver-specific cell types. Our results show that expression of US6 or US11 proteins significantly increased the engraftment of human MSC in the fetal sheep liver, and that donor-derived MSC could still be reprogramed into human hepatocyte-like cells. Since one of the major factors responsible for the failure of allogeneic-donor MSC to engraft is the mismatch of HLA-I molecules between the donor and the recipient [18], [19], [23], [34], MSC-US6 and MSC-US11 could constitute an off-the-shelf product to overcome donor-recipient HLA-I mismatch.

## Results

### HCMV US proteins down-modulate HLA-I expression on MSC

**Figure 1:**
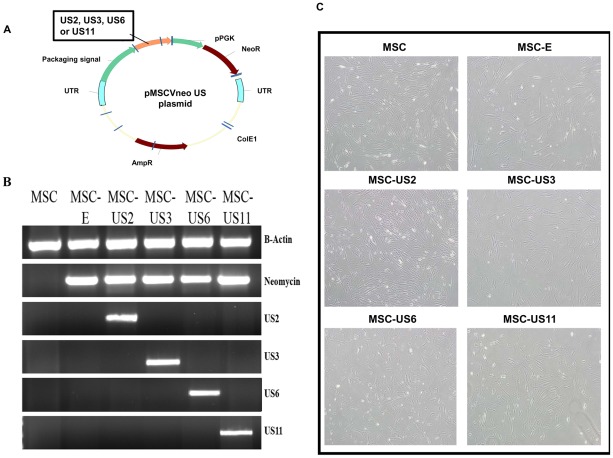
Generation of US-transduced MSC. (A) HCMV US2, US3, US6 and US11 cDNA sequences were cloned into the pMSCVneo plasmid between EcoRI and XhoI or Sal-I. US genes were driven by the MSCV LTR promoter while the Neomycin resistance marker gene (NeoR) was under the control of an internal PGK promoter. (B) Total RNA was extracted from each of the transduced and untransduced MSC populations and, after reverse transcription, cDNAs were obtained and amplified using specific primers for each of the US genes, NeoR and B-Actin. (C) Light microscope image at 10× original magnification of the different MSC populations showing similar morphology during cell culture. Images were captured with an Olympus IX-71 microscope.

MSC were transduced with different MSCVneo retroviral vectors expressing US2, US3, US6 or US11, as described in detail in the material and methods section. Non-transduced MSC, or MSC transduced with an empty MSCVneo retroviral vector (MSC-E) were used as controls (Figure 1A). At 48 hrs after transduction, stable US recombinant MSC were selected with 500 µg/ml G418 for 5 days, replacing the selection media every two/three days. Therefore, only cells that expressed neomycin resistance (NeoR) survived the selection process. Transduced MSC, after selection, were analyzed for expression of the respective US transcripts as well as the presence of the NeoR antibiotic resistance/selection marker. Using PCR and the primers shown in Table 1, we determined that the majority of the transduced cells expressed the corresponding US gene and the antibiotic resistance/selection marker NeoR. Since the expression of each US gene was driven by the MSCVneo 5′LTR, similar transcription levels of different US genes were attained (Figure 1B).

**Table 1:** PCR primers for testing US-transduced and untransduced MSC.

Primer Name	F/R^a^	Sequence (5′ to 3′)	Product sizes (Bp)
US2 F Eco	F	ATA**GAATTC**A-ATGAACAATCTCTGGAAAGC	600
US2 R-Sal I	R	ATT**GTCGAC**-TCAGCACACGAAAAACCG	
US3 F-EcoRI	F	ATA**GAATTC**AA-ATGAAGCCGGTGTTGG	561
US3 R-Xho I	R	GCG**CTCGAG**-TTAAATAAATCGCAGACGG	
US6 F-EcoRI	F	AAG**GAATTC**-ATGGATCTCTTGATACGTCTCGG	552
US6 R-Xho I	R	ATT**CTCGAG**-TCAGGAGCCACAACGTCG	
US11 F-EcoRI	F	TAA**GAATTC**A-ATGAACCTTGTAATGCTT	648
US11 R-Xho I	R	TAT**CTCGAG**-TCACCACTGGTCCG	
Neomycin F	F	GTGGAGAGGCTATTCGGCTA	481
Neomycin R	R	CCTTGAGCCTGGCGAACAGT	
β-actin F	F	ACTCCTGCTTGCTGATCCAC	474
β-actin R	R	TGGCTACAGCTTCACCACC	

a F, Forward Primer; R, Reverse Primer.

**Figure 2:**
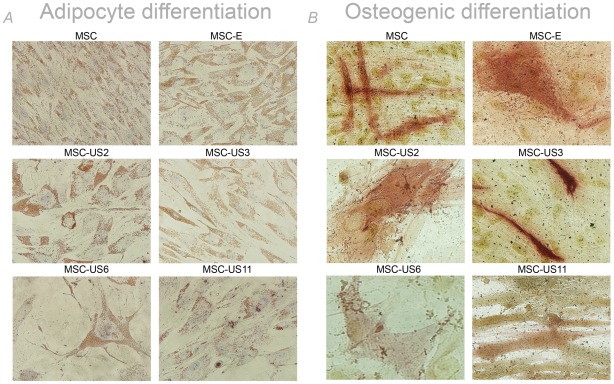
US transduced MSC possess the same differentiative capacities as untransduced MSC. (A) Adipocytic differentiation of transduced and untransduced MSC. Lipid vacuoles were observed by red staining and nuclei by blue staining. (B) Osteogenic differentiation of transduced and untransduced MSC. Red staining indicated alkaline phosphatase activity, blue staining the nuclei, and black staining the calcium deposits. All images were captured with an Olympus IX-71 microscope at 40× original magnification.

The morphology of the transduced MSC remained similar to that of non-transduced MSC (Figure 1C), and all US-transduced cells showed comparable ability to differentiate into adipocytes or osteocytes as untransduced MSC or MSC transduced with empty retrovirus (MSC-E) (Figure 2A and B). Furthermore, all MSC populations remained undifferentiated if not stimulated with external agents and they all continued to express cell surface markers such as CD29, CD90 and CD105.

**Figure 3:**
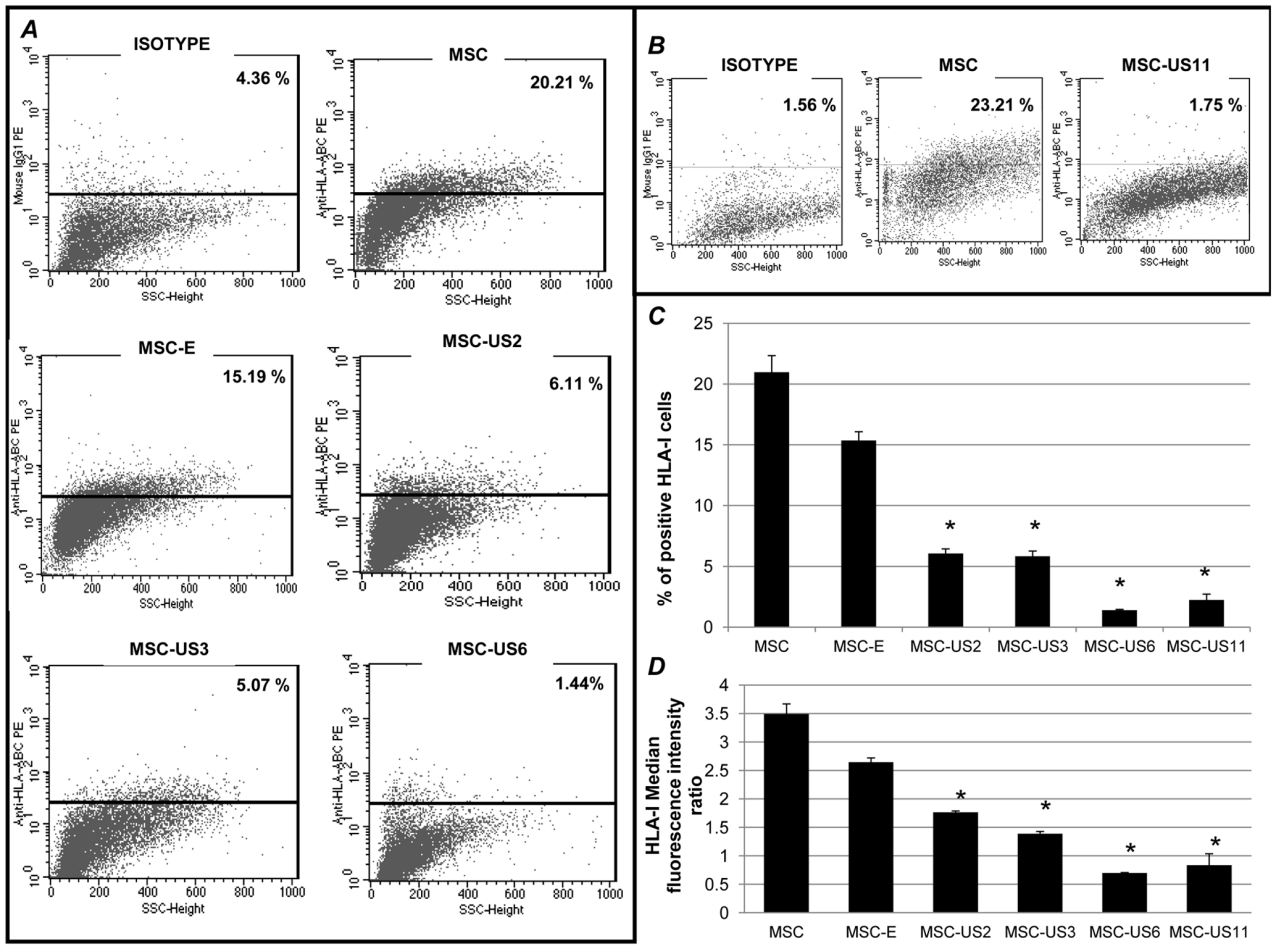
HCMV US proteins down-regulate HLA-I surface expression on MSC. (A) MSC, MSC-E, MSC-US2, MSC-US3 and MSC-US6 were analyzed for HLA-I surface expression by flow cytometry. Panel A shows the results of a representative experiment from at least 3 independent studies. (B) MSC and MSC-US11 were analyzed for HLA-I surface expression by flow cytometry. Panel B shows the results of a representative experiment from at least 3 independent studies. The percentage of HLA-I positive cells was calculated by subtracting background fluorescence as determined by staining with the respective isotype control. (C) Average of the percentage of HLA-I positive cells in 3 independent experiments, and (D) respective Median Fluorescence Intensity ratio as determined by flow cytometry. MFI ratio (MFI cell population/MFI cell population isotype). The results are shown as mean± SEM (* indicates p<0.01 when comparing the transduced MSC with untransduced MSC and MSC-E population).

We then analyzed, by flow cytometry, expression of HLA-I in US-transduced MSC, MSC-E and non-transduced MSC. A representative panel of the results obtained with three independent experiments is shown in Figure 3 A and B, which depicts the percentage of HLA-I positive MSC after subtracting the isotype control. Figure 3C summarizes the percentage of HLA-I positive cells obtained in 3 independent experiments, and Figure 3D shows, for the same experiments, the different Median Fluorescent Intensity (MFI) Ratios for US-transduced MSC, MSC-E and non-transduced MSC. MFI ratio was obtained by dividing HLA-I's MFI by the respective isotype's MFI. Transduction of MSC with MSCVneo empty retroviral vector resulted in a decrease in the percentage of transduced cells expressing HLA-I, from 20.98±1.36% to 15.34±0.73% (p>0.01). A non-statistically significant reduction of the MFI ratio was also observed. Before transduction, overall HLA-I MFI ratio was 3.49±0.17 and after transduction with the empty retroviral vector, the MFI ratio became 2.65±0.07 (p>0.01). However, transduction with recombinant retrovirus expressing either US2 or US3 resulted in a statistically significant reduction in HLA-I levels, when compared to both untransduced MSC or MSC-E. US2 caused a 60% reduction in the percentage of cells expressing HLA-I, (6.05±0.38%) (p<0.01) and a significant reduction in MFI ratio (1.76±0.02) when compared to MSC-E (p<0.01) and untransduced MSC (p<0.01). US3 decreased the percentage of cells expressing HLA-I by 62% (5.83±0.43%) (p<0.01) and a decrease in MFI ratio to 1.39±0.04 (p<0.01). US11 was even more efficient than US2 and US3 in reducing HLA-I expression, and was able to diminish the number of positive cells by 85% when compared with MSC-E (MSC-US11 HLA-I: 2.24±0.47%, (p<0.01); MFI ratio: 0.84±0.2, (p<0.01)). US6 HCMV protein was able to generate the most pronounced reduction, 91%, in HLA-I expression on MSC, when compared to MSC-E, with only 1.40±0.06% (p<0.01) of MSC-US6 expressing HLA-I and a MFI ratio of 0.70±0.006 (p<0.01). When the effect of US6 on percentage of cells expressing HLA-I was compared to that of US2, US3, and US11, a statistically significant difference was found between US6 and the two former transduced MSC (p<0.01), but not the latter (p>0.01). Also, there was not a statistically significant difference between MSC-US6 MFI ratio and MSC-US11 MFI ratio (p<0.01).

### Expression of HCMV US proteins on MSC decreases peripheral blood mononuclear cell (PBMNC) proliferation

**Figure 4:**
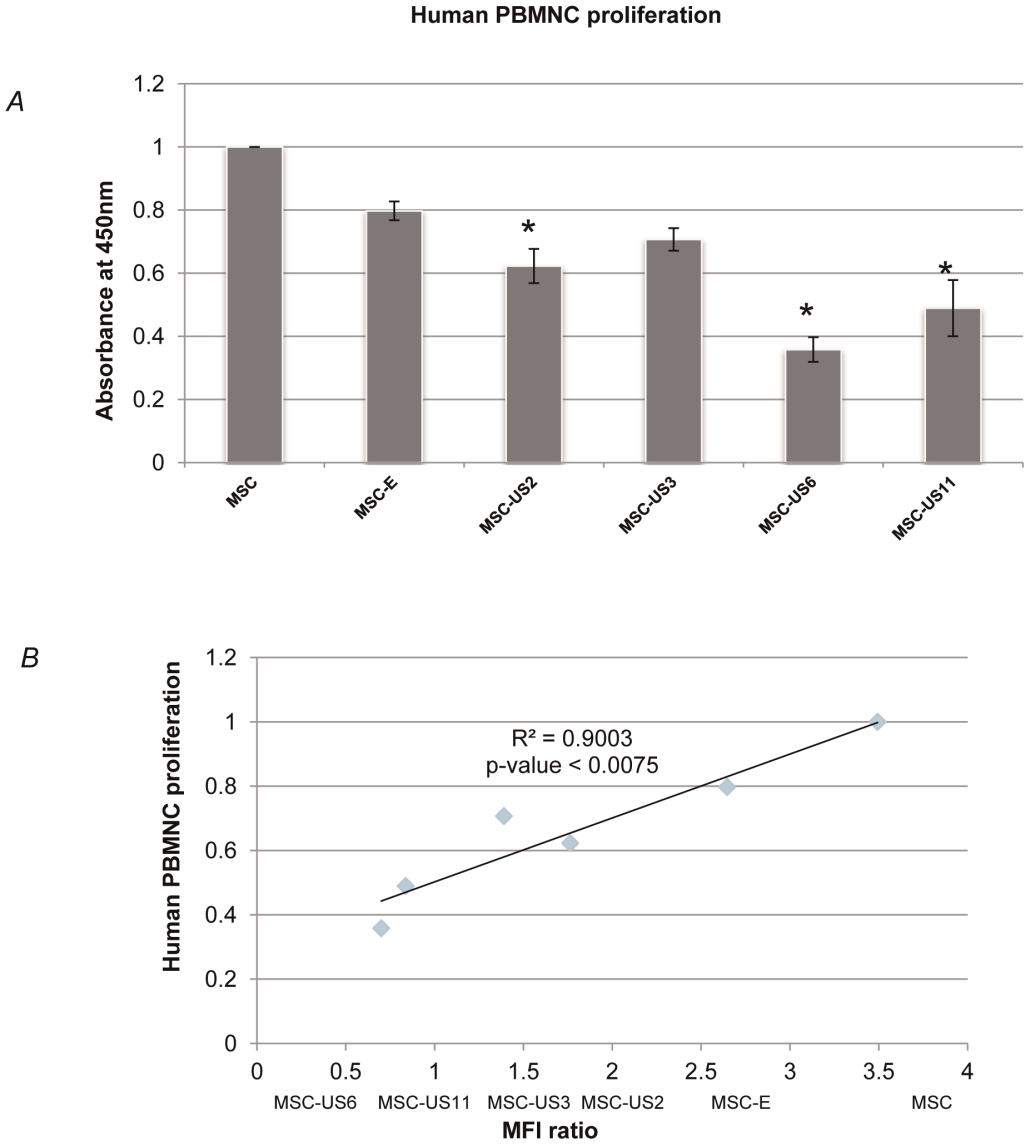
Expression of HCMV US proteins by MSC decreases PBMNC proliferation and correlates with the levels of HLA-I. (A) Each of the transduced and untransduced MSCs were used as stimulators and were co-cultured with responders human PBMNC. After five days, DNA synthesis was assayed with the BrdU cell proliferation colorimetric ELISA. Data represents mean ± SEM of four independent experiments. In each experiment the specific stimulator-responder co-culture was performed in triplicate (* indicates p<0.01 and were considered statistically significant compared to MSC-E levels). (B) Furthermore a direct correlation was found between the levels of HLA-I MFI and human PBMNC proliferation.

Several studies demonstrated that MSC alone are able to suppress PBMNC proliferation [9], [10], [11], [15]; however, recent reports showed that allogeneic MSC can be rejected by CTL and that one of the major factors is the mismatch of HLA class I molecules between the donor and the recipient [18], [19], [23]. Therefore, we hypothesized that the decrease in HLA-I expression induced by US HCMV proteins would lead to reduction of PBMNC proliferation when in the presence of allogeneic MSC. PBMNC proliferation values obtained by co-culturing PBMNC with allogeneic MSC were arbitrarily set as 1. Proliferation values for PBMNC when in the presence of MSC-US and MSC-E were then re-calculated, within the same experiment, by expressing these values as a ratio of the set value of 1 for PBMNC co-cultured with untransduced allogeneic MSC. The results of these experiments (n = 4) are shown in Figure 4A. Although transduction of MSC with the empty retroviral vector caused a decrease in human PBMNC proliferation (MSC vs. MSC-E; 1 vs. 0.80±0.03; p<0.01), this effect was far more pronounced when MSC were transduced with vector encoding US proteins 2, 6 and 11. When compared with MSC-E, the expression of US2 and US3 on MSC reduced human PBMNC proliferation by 22.5% (from 0.80 to 0.62±0.05; p<0.01) and 11% (from 0.80 to 0.71±0.03; p>0.01) respectively, while expression of US11 and US6, decreased PBMNC proliferation by 39% (0.49±0.09; p<0.01) and 55% (0.36±0.04; p<0.01). Furthermore a direct correlation was found between the levels of HLA-I and human PBMNC proliferation (Figure 4B).

Since sheep was used as the *in vivo* model to study engraftment of human MSC transduced with US proteins, we also analyzed the effect of each one of these cell populations on the induction of sheep PBMNC proliferation (Figure S1). The values for sheep PBMNC proliferation were obtained in a similar way to those for human PBMNC proliferation. Transduction of MSC with empty retrovirus reduced, but not significantly, PBMNC proliferation (MSC vs. MSC-E; 1 vs. 0.86±0.07; p>0.01). Also, expression of US2 or US3 did not decrease sheep PBMNC proliferation when compared to MSC or MSC-E (1.01±0.11; 1.05±0.11; respectively) (p>0.01). However, expression of US6 or US11 by MSC significantly reduced sheep PBMNC proliferation when compared to MSC-E, 30% and 27% respectively (0.56±0.06 (p<0.01) and 0.58±0.07 (p≤0.01)).

### Expression of HCMV US proteins protects MSC against NK cell lysis

**Figure 5:**
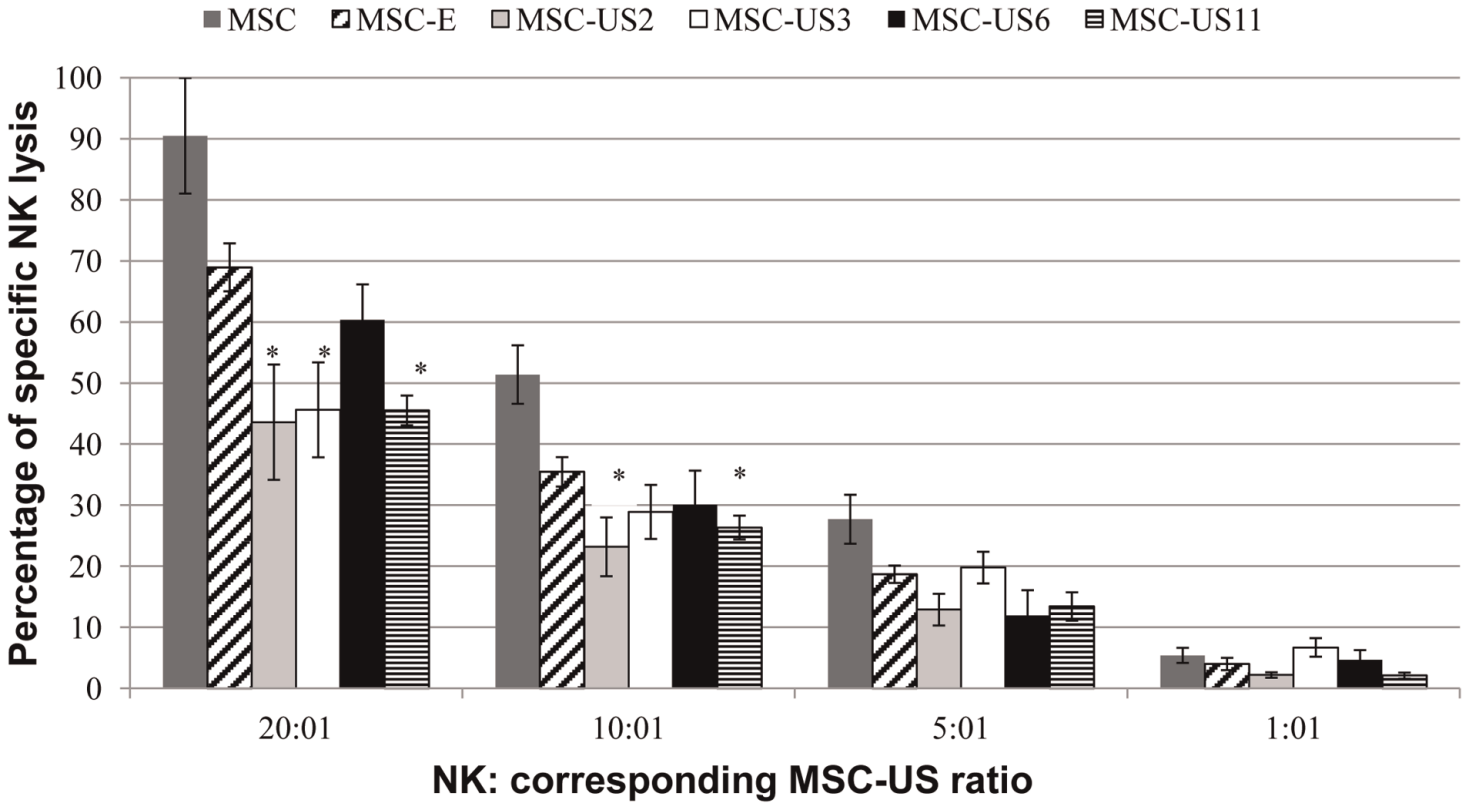
Expression of HCMV US proteins protects MSC against NK cell lysis. Stimulators, MSC-E, MSC-US2, MSC-US3, MSC-US6, MSC-US11 and untransduced MSC were co-cultured independently with different concentrations of NK-92MI cells at 20∶1, 10∶1, 5∶1 and 1∶1 effector∶target ratios for 4 hrs. Release of lactate dehydrogenase was measured after cell lysis by ELISA. The percent specific lysis was calculated for each cell population and effector∶target ratio. The percentage of spontaneous lysis for all of the MSC tested in cytotoxicity assays ranged from 0.3–9.09%. Data represents the mean ± SEM of 6 independent experiments for each ratio between NK92MI cells and each transduced and untransduced cell line. (* indicates statistically significant difference between % specific lysis of US transduced MSC and MSC-E).

There have been controversial reports regarding whether reduction in HLA-I expression by HCMV US proteins renders infected cells more susceptible to NK killing [35], [36]. Therefore, we wanted to determine whether engineering MSC to express lower levels of HLA-I resulted in higher susceptibility of these cells to NK lysis. We thus analyzed the effect of expressing the HCMV US proteins on NK cell recognition, activation and killing of MSC. NK cell cytotoxicity assays (n = 6) were performed as described in the material and methods section, and the results are depicted in Figure 5. NK killing assays were performed using the interleukin-2 independent cell line NK-92MI. This cell line, derived from NK-92, was used due to its high proliferation rate in cell culture, and high cytotoxic properties in the absence of IL2. The NK-92MI cell line is similar to activated NK cells in terms of surface receptor expression and functional properties, but it lacks expression of the KIR receptor of the p58 complex, which is an inhibitory receptor for HLA-C [37], [38]. Analysis of HLA-C surface levels showed that the percentage of positive cells expressing HLA-C was almost neglible, 1.3±0.2% in untransduced MSC, and 0.7±0.2% and 0.7±0.3% for MSC-US6 or MSC-US11 respectively. Therefore, the interaction between inhibitory receptor KIR and MSC, independently of using primary NK cells or NK-92MI, does not occur due to absence of HLA-C on MSC. NK-92MI also lacks the CD16 receptor, which is required for antibody-dependent cellular cytotoxicity [37], [38]. Since the NK killing assays performed in this study did not require antibody-dependent cellular cytotoxicity activity of the NK cells, the NK-92MI cell line constituted an appropriate tool for testing susceptibility of US-transduced MSC to NK cytolysis.

Results from NK killing assays demonstrated that transduction with an empty retrovirus did not result in statistically significant reduction of NK killing-mediated lysis of MSC, when compared to untransduced MSC for most of the ratios NK∶MSC analyzed (Figure 5). At a 20∶1 effector∶target (NK92-MI∶MSC) ratio, the percentage of specific lysis of untransduced MSC was 90.50±9.5% and of MSC-E 68.93±3.92% (p>0.05). Between MSC expressing US11 and US3, NK cell-mediated lysis was similar, and 34% lower than MSC-E (p<0.05). In MSC-US2, NK cell-mediated lysis was 37% less than that of MSC-E. NK killing-mediated lysis of MSC-US2 was 43.59±9.43% (p<0.05), that of MSC-US3 was 45.60±7.76% (p<0.05), and that of MSC-US11 was 45.52±2.43% (p<0.05). Furthermore, at this ratio of 20∶1, expression of US6 on MSC did not result in a significant reduction of NK killing (60.37±5.77) when compared to MSC-E. When the effector∶target ratio was 10∶1, US2 expression reduced NK cell lysis by 35% and US11 by 26%, when compared to MSC-E (% specific lysis: 23.19±4.82% ( p<0.05); 26.33±1.95% ( p<0.05) vs. 35.45±2.40%). However, at this ratio, neither MSC-US3 (28.90±4.4%) nor MSC-US6 (30.10±5.6%) produced a statistically significant reduction in NK–mediated lysis compared to MSC-E. At a ratio of 5∶1, the ability of NK-92MI cells to target all of the US transduced MSC was not statistically different from MSC-E; however, at this ratio, MSC-US2, MSC-US6 and MSC-US11 had a statistically significant reduced NK–cell mediated lysis compared to untransduced MSC (Figure 5). At a ratio of 1∶1 the overall ability of NK-92MI cells to target MSC was similar amongst all the MSC tested.

These results show that the decrease in HLA-I expression following the transduction of MSC with retroviral vectors encoding US6 or US11 does not increase the ability of NK cells to target these cells as compared to normal MSC. Moreover, at E∶T ratios higher than 5∶1, expression of US proteins on MSC, in particular US11, led to an increased protection of MSC against NK cell lysis.

### HLA-E and HLA-G1 expression levels are similar between cells expressing US6 or US11 and the MSC-E cell line

Since we showed that US6 and US11 protected MSC from NK killing despite the downregulation of HLA class I expression, we next investigated which molecules could be mediating transduced MSC protection from NK lysis. HLA-E is a non-classical HLA-I molecule, and a specific ligand for the NK cell inhibitory receptor CD94/NKG2. Although it has been reported that during CMV infection HLA-E expression is not affected by HCMV US11 or US6 proteins [31], we set out to determine whether the same held true when these individual HCMV proteins were intentionally over-expressed on MSC. Therefore, using flow cytometric analysis, we investigated whether HLA-E expression on MSC-US6 or MSC-US11 could be mediating protection against NK lysis. The percentage of positive HLA-E cells for non-transduced MSC was almost negligible (0.35±0.03%, MFI ratio: 0.84±0.02). Furthermore, the percentage of positive cells or MFI ratio for HLA-E expression did not change after expressing US6 (% of MSC-US6 0.167±0.003%, MFI ratio 1.00±0.06) or US11 proteins (MSC-US11 0.51±0.12%, MFI ratio 0.922±0.007).

Next, we studied expression of HLA-G1, another non-classical HLA-I molecule, whose interaction with inhibitory receptors found on NK cells, such as ILT2 and ILT4 leads to the inhibition of NK- and T-cell activation programs. Studies using flow cytometry demonstrated that untransduced MSC expressed HLA-G1 protein constitutively (MFI ratio: 1.36±0.03; 11.53±0.7%) and that transduction of MSC with empty retrovirus or retrovirus expressing US11 protein did not significantly modify surface levels of HLA-G1 (MFI ratio: 1.83±0.02 for MSC-E; 1.43±0.13 for MSC-US11), but increased the percentage of cells that were expressing HLA-G1. The percentage of MSC-US11 positive cells for HLA-G1 was 17.05±1.65%, similar to that of MSC-E (18.79±1.71%). The percentage of MSC-US6 expressing HLA-G1 was less than for all the other cells (6.45±0.97%) (p<0.05), but a slight increase in HLA-G1 surface levels was seen when compared to those of MSC-E (MFI ratio: 2.31±0.29 vs. 1.83±0.02) (p>0.05).

### Expression levels of several NK activating ligands are similar between MSC-US6, MSC-US11 and MSC-E

Since the cytolytic capability of NK cells depends on the balance between activating and inhibitory signals on the target cell, we next investigated whether there were differences in the expression of known activating NK cell ligands on the surface of untransduced MSC, MSC-US6, and MSC-US11 (n = 3). Using antibodies specific for the NKG2D ligands, ULBP 1, 2 and 3, we were able to determine, in 3 different experiments, that the percentage of MSC, MSC-US6, and MSC-US11 expressing these molecules was almost negligible. Only 0.5±0.2% of MSC expressed ULBP1, 0.08±0.02% ULBP2, and ULBP3 was absent on MSC. In similarity, only a very small percentage of MSC-US6 and MSC-US11 expressed ULBP1 (0.8±0.07 and 1.9±0.02 respectively) and ULBP2 (0.1±0.01 and 0.07±0.02), and none expressed ULBP3.

We also studied other NKG2D ligands MICA/B (n = 3), and only 1.7±0.06% of MSC, 3.3±0.1% of MSC-US6 and 1.7±0.03 of MSC-US11 were positive for these molecules, demonstrating that these ligands do not play an important role in the MSC/NK interaction.

We next looked for the presence of the DNAM-1 ligands, CD112 and CD155, on the surface of the different MSC populations. Flow cytometric analysis demonstrated that both transduced and untransduced MSC were negative for CD112. By contrast, CD155 was expressed on 18.1%±2.1 of untransduced MSC (MFI ratio: 4.69±0.07), on 21.99%±0.96 of MSC-E (MFI ratio:4.24±0.05), and on 22.1±0.8% of the MSC-US11 (MFI ratio:4.25±0.09). A higher percentage of MSC-US6 expressed CD155 (37±0.7%) when compared to both MSC and MSC-E (p<0.05). The MFI ratio of MSC-US6 was also increased when compared to the same populations (5.36±0.08) (p<0.05).

Since it has also been reported that the ability of NK to kill CMV-infected cells depends on the elevated levels of expression of CD58 on the surface of infected cells and not on the down-regulation of cell surface Class I HLA [35], we also investigated whether transducing MSC to express US6 and US11 resulted in modification of CD58 expression on cell surface. CD58 was not expressed by untransduced MSC, MSC-US6 or MSC-US11 (data not shown).

### HLA-II expression on MSC is not induced by HCMV US6 or US11 proteins

Although MSC do not usually express HLA-II molecules [9], several studies have shown that when these cells are exposed to certain growth factors [39] or pro-inflammatory cytokines such as Interferon-gamma [40], induction of HLA-II occurs in MSC. Therefore, we next examined the effect of forced expression of US6 or US11 proteins on HLA-II regulation within MSC. As determined by flow cytometric analysis, transduction of MSC with MSCVneo retroviral vectors over-expressing US6 or US11 did not cause a statistically significant difference in HLA-II expression when compared with control MSC. As previously reported, MSC did not express HLA-II, with HLA-II MFI not differing from that of the isotype control (MFI ratio:1.30±0.14). In similarity, the levels of HLA-II expression, as determined by MFI ratio, on MSC-US11 (MFI ratio: 1.06±0.01) and MSC-US6 (MFI ratio:1.23±0.04), were similar to or lower than the MFI ratio of non-transduced MSC, demonstrating that up-regulation of HLA-II did not occur after engineering of the MSC with US proteins.

### Transplantation of human MSC-US6 and MSC-US11 in fetal sheep results in higher levels of liver cell engraftment when compared with MSC-E as determined by quantitative PCR (qPCR)

We have previously shown that human MSC engraft in the liver of fetal sheep after in utero transplantation [5], [32]. Therefore we tested the ability of transduced cells that had provided the best immunological results *in vitro*, MSC-US6 and MSC-US11, to engraft and generate hepatocyte-like cells, and compared them to control MSC-E. To this end, we transplanted 61 day-old pre-immune fetal sheep recipients (n = 6) via intraperitoneal route with MSC-US6, MSC-US11, or MSC-E and compared their levels and patterns of engraftment. At 127 days of gestation (term of 145 days), animals were euthanized and analyzed for liver engraftment.

**Figure 6:**
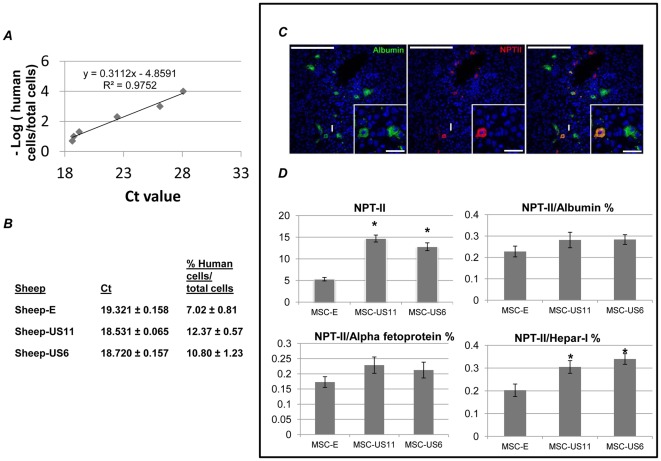
Quantification of Human MSC engraftment in fetal sheep liver. (A) Standard curve for human GAPDH amplification. Different Ct values were obtained by amplification of GAPDH at different percentages of human MSC/(human MSC+sheep MSC cells) concentrations. qPCR reaction for each concentration was repeated three times. (B) Percentage of total human cells engrafted in fetal liver of sheep that were injected with different MSC. The results are mean ± SEM of twelve replicates of qPCR reactions for MSC-E and MSC-US6 transplanted animals or six replicates of qPCR reactions for MSC-US11 transplanted animals. (*C*) Representative immunofluorescence staining and confocal microscopy of NPT-II and albumin staining of liver sections from sheep receiving MSC-US6 cell line. Images were captured using a *Fluoview 1000* confocal microscope with a 40× objective. The white arrow marks the area of the inset. Scale bars: 50 µm main picture; 12.5 µm on inset *(D) (top)* on the left, quantification of total NPT-II positive cells in fetal liver of sheep that were injected with the different MSC cell lines. On the top right, quantification of dual NPT-II and albumin positive cells in fetal liver of animals that were injected with the different MSC cell lines. On the bottom left, quantification of dual NPT-II and alpha-fetoprotein positive cells in fetal liver of sections from animals that were injected with the different MSC. On the bottom right, quantification of dual NPT-II and Hepar-I positive cells in fetal liver of sheep that were injected with the different MSC cell lines. The results are means ± SEM of at least 90,000 cells that were counted for NPT-II staining within the fetal liver sections from sheep that were injected with the different MSC. * p<0.05.

In order to determine the exact percentage of human MSC engraftment within the liver of the sheep that received each of the different MSC-US cells, we performed human-specific qPCR as described in detail the Material and Methods section. First, as shown in Figure 6A we constructed a calibration curve using different ratios of human MSC/total cells (0.0001, 0.001, 0.005, 0.01, 0.05, 0.1, and 0.2), and their corresponding Ct values (28.076, 26.093, 22.449, 19.218, 18.759, and 18.637). We then performed qPCR using DNA extracted from the livers of the transplanted animals and GAPDH human specific-primers. We used the obtained Ct values to extrapolate the percentage of human MSC engraftment within the liver sample from each of the MSC-transplanted animals. As can be seen in Figure 6B, fetal liver from sheep injected with MSC-E cells exhibited human MSC engraftment levels of 7.02±0.81%. In the animals that received MSC expressing US6 protein, the levels of human MSC engraftment were 10.80±1.23%, corresponding to a 1.5-fold increase in engraftment (p<0.05). In similarity, MSC-US11 had engraftment levels of 12.37±0.57% within the fetal liver, corresponding to a 1.8-fold increase over the engraftment seen with MSC-E (p<0.05). There was no significant difference in levels of engraftment within the fetal sheep liver when comparing cells expressing US6 to those expressing US11 (p>0.05). These results demonstrate that forced expression of US6 or US11 on human MSC increased their ability to engraft within the fetal sheep liver following in utero transplantation. Another possible explanation it would be that MSC-US6 or -US11 upon engraftment proliferated more than the MSC-E control. Although MSC-US6 or -US11 did not show increased proliferation rates in vitro (data not shown), it is also possible, but not likely, that higher levels of engraftment were due to higher MSC-US6 or -US11 cell proliferation upon in vivo lodging.

### Immunofluorescence analysis with an anti-neomycin phosphotransferase II antibody confirms enhanced engraftment of MSC-US11 and MSC-US6 in fetal sheep liver

Human MSC engraftment within the liver of the transplanted animals was also analyzed by using an antibody against the vector-encoded marker, neomycin phosphotransferase II (NPT-II). Liver samples collected from sheep that received the various MSC lines were cryopreserved, and processed as described in the Materials and Methods section. Engrafted human MSC were localized and quantified by staining with an antibody to the NPT-II marker gene contained within the MSCV vector, as shown in figure 6C (middle panel) and D. NPT-II staining on liver from fetal sheep that received MSC-E revealed 5.3±0.4% NPT-II positive cells. In animals that received MSC-US6 and MSC-US11, the percentages of NPT-II+ cells were 12.8±0.9% and 14.7±0.8%, respectively, which corresponds to a 2.4 and 2.8-fold increase in engraftment over that achieved with MSC-E. The enhanced engraftment produced by US proteins was, in both cases, statistically significant compared to the engraftment produced by MSC-E (p<0.01). In similarity to the results obtained with qPCR, the difference in engraftment capabilities of MSC-US6 and MSC-US11, was not statistically significant (p>0.05), but in both analyses animals receiving MSC-US11 had higher levels of engraftment.

### MSC expressing US HCMV proteins are able to differentiate in vivo in hepatocyte-like cells after engraftment within the fetal liver

Since we have previously shown that transplantation of human MSC into fetal sheep also results in formation of donor-derived hepatocytes [5], [32], here we investigated whether modifying MSC with the US proteins would alter the differentiative capacity of these engrafted MSC. Therefore, we performed dual immunofluorescence staining for NPT-II and albumin. NPT-II staining unequivocally identified the human MSC that had engrafted within the sheep liver, as described above, while albumin labeling identified both human and sheep albumin-producing hepatocytes (Figure 6C left panel). As can be seen shown in Figure 6C (right panel), human MSC reprogramed to a hepatocyte-like phenotype, as evidenced by colocalization of NPT-II and albumin staining. Quantitation of the percentage of NPT-II/albumin-positive cells within liver sections from fetal sheep that had received each of the three different human MSC lines is shown in Figure 6D. MSC-E, MSC-US11 and MSC-US6 generated 0.23±0.02%, 0.28±0.04% and 0.28±0.02% of NPT-II/albumin-positive cells respectively.

Analysis of NPT-II and alpha-fetoprotein dual-positive cells, demonstrated that transduced MSC engrafted and generated primitive hepatocyte-like cells. Specifically, in the presence of US6 or US11 protein, 0.21±0.03% and 0.23±0.03% of the liver cells were also NPT-II-Alpha fetoprotein-positive, respectively. MSC-E generated less NPT-II-Alpha fetoprotein-positive cells (0.17±0.02%) when compared to that of either the MSC-US6 or MSC-US11 (Figure 6 D).

We next analyzed liver sections from sheep transplanted with the different recombinant MSC using an anti-Hepar-I antibody. Quantification of dual-positive cells in liver sections from animals transplanted with MSC-E cells showed that 0.20±0.03% of the cells were NPT-II+, Hepar+ (Figure 6D). In animals transplanted with MSC-US6 protein, 0.34±0.02% liver cells were dual-positive for NPT-II and Hepar-I (p<0.05 compared to MSC-E engraftment), and similarly, following transplantation with cells expressing US11, 0.30±0.03% of the cells within the recipient liver were expressing both markers (p<0.05 compared to MSC-E engraftment). However, there was not a statistically significant difference in the percentage of Hepar positive cells between MSC-US6 and MSC-US11 (p>0.05).

## Discussion

Despite numerous studies reporting the efficacy of allogeneic MSC as therapeutic tools (reviewed in [41]), controversy still remains whether these cells, upon transplantation, are able to elicit an immune response in the recipient, lessening their therapeutic potential when compared with their autologous counterpart. MSC, regardless of graft source, possess the same immunomodulatory and anti-inflammatory properties. This is due, at least in part, to the antiproliferative effects of MSC on immune and other somatic cells, leading to arrest of cell division in T and NK cells, and thwarting cytokine secretion and cytotoxicity [42]. Still, allogeneic MSC are not invisible to the immune system, and several studies have demonstrated that administration of allogeneic MSC induced immunogenic responses, and that MSC were recognized and rejected by the immune system [2], [18], [19], [43]. Since MSC express negligible amounts of HLA-II, but display variable levels of HLA-I on their surface, and harbor several ligands to activating NK cell receptors [44], [45], it is likely that MSC can become a target of NK and CTL.

Here, we genetically engineered MSC to express the HCMV proteins US2, US3, US6 and US11, since these proteins were previously shown to reduce HLA-I surface levels on somatic cells. Moreover, this reduction in HLA-I levels prevented CTL recognition, activation and killing of infected cells during a normal HCMV infection [30], [31], and of breast and neuronal cells when these cells were transfected with these HCMV US genes and exposed to IFN-gamma [46], [47]. In agreement with these studies, our results show that MSC transduced with retroviral vectors expressing HCMV proteins US2, US6 and US11 reduced levels of HLA-I on their surface, when compared with non-transduced MSC, or with MSC transduced with a retroviral vector encoding only the NeoR gene. Furthermore, we demonstrated that US6 and US11 were the most efficient in downregulating expression of HLA-I and therefore reducing MSC immunogenicity, as demonstrated by the correlation between HLA-I expression and the decrease in human PBMNC proliferation using MLR assays. MSC-US6 produced the greatest reduction of HLA-I expression, decreasing MFI by 90%, and the most pronounced decrease in human (61%) PBMNC stimulation as well as sheep (35%) PBMNC stimulation. The differences in the ability of the various US proteins to reduce HLA-I expression on MSC are in agreement with previous studies using other cell types [48], [49], and are most likely due to the distinctive mechanism by which each of the proteins are known to down-regulate MHC-I molecules [50], [51], [52]. Furthermore, they are also in conformity with what has been reported by others demonstrating that US11 is more effective in degrading class I molecules [53], [54].

Because reduction of HLA-I molecules usually renders cells susceptible to NK lysis, and since in an allograft setting NK cells are frequently recruited to the site of the transplant and contribute to graft rejection [55], [56], it was our concern that down-regulation of HLA-I expression on MSC would lead to an increased susceptibility of MSC to NK lysis. Here we show that the expression of US proteins on MSC, and consequent HLA-I down-regulation, did not increase their susceptibility to NK killing, and that US2, US3 and especially US11-transduced MSC were protected from NK cytotoxic effects at certain NK: MSC ratios. Although MSC used in this work were not of adult origin, and it has been reported that fetal and adult MSC differ in their interactions with NK cells, both fetal and adult MSC are susceptible to lysis by activated NK cells [45]; therefore, we anticipate that the transduction of adult MSC with US proteins 2, 3, or 11 could also be successfully used in adult MSC to avoid CTL and NK activation and decrease killing of transplanted allogeneic cells.

In order to start dissecting possible mechanisms by which US proteins enable NK killing evasion, we studied the surface levels of several activating (ULBP 1, 2 and 3, MICA/B, CD112 and CD155) and inhibitory ligands (HLA-E and HLA-G1), as well as CD58 molecules (higher levels of which are linked to enhanced NK killing), on all the MSC populations. HLA-E, a non-classical HLA-I molecule and a specific ligand for the NK cell inhibitory receptor CD94/NKG2, was expressed at very low levels or absent in all of the MSC populations, and the same was found for the activating NKG2D ligands ULBP1–3 and MICA/B, the DNAM-1 ligand CD112, and CD58. Therefore, none of these molecules seem to play a role in this particular case of MSC/NK interaction. The absence of these stimulatory molecules provides a possible explanation for the lack of NK killing in the absence of HLA-I expression. Prior studies have shown that even though many circulating cells lack inhibitory receptors, they do not attack normal tissues [57]. Likewise, red blood cells [58] and neural stem cells [59] are not lysed by NK cells, despite expressing no HLA-I. This is presumably due to the requirement for activating signals to initiate NK lysis. This is agreement to what has been reported for bone marrow derived MSC, in which CD112, MICA/B, and ULBP1–3 have been shown to be expressed and to enable NK-mediated lysis [11].

HLA-G1, another non-classical HLA-I molecule, has been shown to be constitutively expressed by MSC [60], [61], [62], and interaction of this molecule with the NK inhibitory receptors ILT2 and ILT4, leads to the inhibition of NK- and T-cell activation programs [63], [64]. Our results also showed that US-transduced MSC cell lines expressed HLA-G1 protein at similar levels to MSC-E. Analysis of CD155, a DNAM-1 activating ligand, revealed that MSC constitutively express the marker, and that CD155 expression was up-regulated on MSC-US6 when compared to MSC, MSC-E and MSC-US11. Still, MSC-US6 were as susceptible to NK killing as MSC-E and no more so than untransduced MSC, even though MHC-I surface molecules on MSC-US6 were significantly reduced when compared to both cell populations.

Because NK killing assays were performed using the interleukin-2 independent cell line NK-92MI and not primary cells, it could be possible that different results would be obtained if we had used the latter. However, this is highly unlikely, since NK-92MI was used due to its high cytotoxic properties in the absence of IL2, and because MSC lack the ligands for the few receptors that are different between primary cells and NK-92MI. Also, the results presented here demonstrating that, at low NK-to-MSC ratios, the ability of NK to kill both transduced and untransduced MSC is diminished, are in agreement with others that showed this same effect, and that both IFN-gamma and IL-10 were involved in the mechanism of regulating NK function [65]. However, this mechanism does not explain the decrease in NK cytotoxicity towards MSC transduced with US 2,3 and 11 at ratios of 20∶1 and 10∶1, and does not concur with a study which demonstrated that down-regulation of MHC-I expression rendered the cells susceptible to NK cells when transplanted in a xenograft model [66]. However, since our results were obtained with *in vitro* assays and in the other study the results were obtained after *in vivo* transplantation, it is difficult to directly compare the seemingly conflicting results.

Therefore, we used human hepatocyte formation in fetal sheep as a paradigm to investigate whether MSC-US6, that confers higher protection against CTL proliferation but same NK killing protection as MSC-E; and MSC-US11, that grants higher protection against both CTL proliferation and NK killing compared to MSC-E cell line, would allow higher levels of engraftment and differentiation into liver-specific cell types when compared to MSC-E. Analysis of hepatic engraftment of MSC expressing US6 or US11 proteins using PCR and immunofluorescence for the vector-encoded NeoR gene product, NPT-II, revealed that over-expression of US6 and US11, regardless of the difference in NK killing protection, resulted in higher levels of engraftment when compared to MSC-E. Expression of US6 and US11 resulted in a 1.5- and 1.8-fold increase in the levels of liver engraftment, respectively when compared to MSC-E.

Moreover, transduction of MSC with US6 and US11 did not result in alteration of MSC reprograming into hepatocyte-like cells as we had previously described as assessed by immunostaining with antibodies against human albumin, HEPAR-I and alpha-fetoprotein.

In conclusion, we found a potential strategy that allows MSC to evade immunological rejection in mismatched recipients. This approach maximized MSC engraftment in a xenogeneic model and could, in an allogeneic transplantation setting, allow curative numbers of cells to engraft and fulfill their role at the site of injury thereby improving their therapeutic benefit.

## Materials and Methods

### Mesenchymal Stem Cells

Mesenchymal stem cells were obtained from at least 5 different donors as previously described [8] using anti-Stro-1 antibody (R&D Systems, Minneapolis, MN) and magnetic cell sorting (Miltenyi Biotec, Inc., Auburn, CA) to isolate Stro-1+ cells. Stro-1+ cells were then cultured in gelatin (Sigma, St Louis MO) coated flasks and MSCGM™ (Mesenchymal Stem Cell Growth Medium BulletKit®, Lonza, Walkersville, Maryland, USA).

NK 92-MI cells were obtained from American Type Culture Collection (ATCC, Rockville, MD), and were maintained in Alpha MEM (Invitrogen, Carlsbad, CA) supplemented with 2 mM L-glutamine (Invitrogen) 1.5 g/l sodium bicarbonate, 0.2 mM inositol, 0.1 mM 2-mercaptoethanol, and 0.02 mM folic acid, (all from Sigma), 12.5% FBS (Atlanta biologicals), and 12.5% horse serum (Atlanta biologicals).

### Construction of recombinant vectors

US2, US3, US6, and US11 DNA sequences were PCR amplified from a clinical HCMV isolate (kindly provided by Dr. Stephen St. Jeor, University of Nevada, Reno, Nevada, USA), introducing *EcoRI* and *XhoI* restriction sites for US3, US6 and US11 and *EcoRI* and *SalI* for US2 (primer sequences are shown in Table 1). The purified PCR products were then ligated into the pMSCV-Neo retroviral vector backbone (Clontech, Mountain View, CA) that had been previously digested with *EcoRI* and *XhoI*. These recombinant plasmids were transformed into One Shot Top10 chemically competent cells (Invitrogen), and transformed Top10 cells were selected with ampicillin 50 µg/ml (Sigma). Positive clones were confirmed by PCR using primers for the US insert, miniprep digestions and sequencing. For US3, US6 and US11 cloning, digestion was performed with *EcoRI* and *XhoI* restriction enzymes. For US2 cloning, *EcoRI* and *BglII* restriction sites were digested, as US2 cloning generated a SalI/XhoI hybrid restriction site.

### Establishment of retrovirus-producing cell lines

Each US recombinant plasmid and an empty plasmid were transfected into the RetroPack™ PT67 Packaging Cell Line (Clontech) using Lipofectamine 2000 (Invitrogen) according to the manufacturer's instructions. Stable transfectants were selected for 5 days with 500 µg/ml G418 (Fisher), starting at 72 hrs after the transfection. Supernatants were collected and filtered with 0.2 µm low binding protein-syringe filters (Pall Corporation, Ann Arbor, MI).

### Transduction of MSC

Subconfluent cultures of MSC were transduced for 6 hours with filtered supernatant containing either US recombinant or empty MSCVneo retrovirus diluted in serum-free QBSF60 medium (Quality Biological, Gaithersburg, MD) and 8 µg/mL protamine sulfate (Calbiochem, San Diego, CA). After transduction, cell layers were washed and media was changed to MSCGM™ (Lonza). At 48 hrs after transduction, stable US-recombinant MSC were selected with 500 µg/ml G418 (Fisher Scientific, Rochester, NY) for 5 days, replacing the selection media every two/three days. Therefore, after the antibiotic selection all the cells were transduced and consequently expressing NeoR and the corresponding US HCMV gene. Transduction of MSC with empty retrovirus or retroviruses coding for US genes were performed several times with similar efficiency. Stably transduced cells were analyzed for transgene expression and designated as MSC-US2, MSC-US3, MSC-US6 and MSC-US11 according to the HCMV US protein that they expressed, or the lack thereof (MSC-E). Transmitted light images of transduced and untransduced MSC populations were captured with an Olympus IX-71 microscope with a 10× and 40× objective. Also, total RNA was purified from each of the transduced and untransduced MSC using TRIzol® Reagent with the PureLink™ RNA Micro Kit (Invitrogen) and DNase-treated with RQ1 RNase-Free DNase (Promega, Madison WI). In order to analyze presence/absence of NeoR and the corresponding US gene expression, 200 ng of each RNA sample was used for cDNA synthesis using the SuperScript III first-strand synthesis system and random primers (Invitrogen) according to the manufacturer's protocol. For PCR, the reaction mixture consisted of 1.5 mM MgCl_2_, 20 mM Tris-HCl (pH 8.3), 50 mM KCl, 0.2 mM dNTP mixture, 0.02 U of Taq DNA recombinant polymerase (Invitrogen), 0.004 µg/µl of each primer (sequences shown in Table 1) and 5 µl of cDNA. The amplification conditions were initial denaturation at 94°C (3 minutes), followed by 30 cycles of denaturation at 94°C (45 seconds), annealing at 55°C (30 seconds), elongation at 72°C (1 minute 30 seconds), and a final elongation of 10 minutes at 72°C. The PCR products were electrophoresed on a 1% agarose gel in 1× Tris-acetate-EDTA buffer visualized by UV transillumination (Biospectrum) and recorded using visionWorksLS software.

### Adipogenic and osteogenic differentiation of transduced and untransduced MSC

MSC, MSC-E, MSC-US2, MSC-US3, MSC-US6 and MSC-US11 were induced into adipocytes and osteocytes as previously described [5]. After 10 days in culture adipocytic induced cells were fixed with 10% formalin and 60% ethanol and stained with Oil Red O 0.5% solution (Poly Scientific R & D Corp, Bay Shore, NY) for 5 min and Hematoxylin (Sigma) for 10 min. Lipid vacuoles were observed by red staining and nuclei by blue staining. Cells induced to a osteocyte phenotype were fixed after 2 weeks in culture with 95% ethanol and stained with alkaline phosphatase (Sigma) and Fast Violet B solutions (Sigma) for 2 hours at RT and hematoxylin for 10 minutes. Red staining indicated alkaline phosphatase activity and blue staining the nuclei. Calcium deposits were stained with 2.5% silver nitrate for 60 min at RT and finally with sodium carbonate formaldehyde solution for 2 minutes. Calcium deposits were visualized as black spots.

### Flow cytometry analysis

MSC, MSC-E, MSC-US2, MSC-US3, MSC-US6 and MSC-US11, prior to confluency, were detached by cell scraping in 1× PBS, in order to preserve extracellular epitopes. Cells were then counted and their viability checked using Trypan Blue reagent (Amresco, Solon OH). All the cells were more than 99% viable. 1×10^5^ cells were incubated for 15 min at RT with mouse anti-human HLA-ABC PE (Becton Dickinson, Pharmingen, San Jose, CA) or mouse IgG1 PE [clone G46-2.6] (BD Bioscience, San Jose, CA) as isotype control. Moreover, MSC, MSC-US6 and MSC-US11 cells were stained with mouse anti-human HLA-E [clone MEM-E/08] (Abcam, Cambridge, MA), mouse anti-human HLA-G FITC [clone MEM-G/9](ABD Serotec) and mouse anti-human HLA DR, DP, DQ FITC [clone TÜ39] (BD Bioscience). The same cell lines were stained with the appropriate isotype control Ab: goat anti-mouse IgG FITC (BD Bioscience) for HLA E staining, mouse IgG1 FITC (ABD Serotec) for HLA-G staining, and mouse IgG2a FITC (AbD Serotec) for HLA class II staining. To test mesenchymal markers on US transduced and untransduced cells, we used mouse anti-human CD29 FITC (Biosource) [clone B-D15), mouse anti-human CD105: FITC (ABD Serotec) (Clone SN6) and mouse anti-human CD90 FITC (BD Pharmingen) [clone 5E10]. Isotype control for CD29 staining was mouse IgG2a FITC (BD Bioscience) and isotype controls for CD90 and CD105 staining were mouse IgG1 FITC (R&D systems). Other antibodies used were: anti-human CD155 FITC, CD112 and MICA/MICB FITC (AbD Serotec, Oxford, UK); anti-human ULBP-1 PE, ULBP-2 PE, and ULBP-3 (R&D Systems Inc., Minneapolis, Minnesota, USA) and anti-human CD58 PE (BD Biosciences, San Jose, US). Secondary antibody used for CD112, and ULBP3 antibodies was goat anti mouse IgG FITC (BD Biosciences, San Jose, US). In all the cases, viable cells were incubated for 15 min with each antibody, washed, fixed with 0.1% azide in PBS, centrifuged and fixed with 1% formaldehyde. A FACSort system (Becton Dickinson) with CELLQUEST software (Becton Dickinson) was used to analyze stained cells. Forward and side-scatter plots were used to exclude the few dead cells and debris from the histogram analysis plots.

### Preparation of Peripheral Blood Mononuclear Cells (PBMNC)

Human PBMNC were prepared from freshly collected, heparinized whole blood samples from different individuals, kindly provided by United Blood Services (Sparks Center, NV). Sheep PBMNC were isolated from different whole blood animal samples. Whole human or sheep blood was diluted 1∶2 with IMDM and mononuclear cells were obtained by performing Ficoll-Hypaque density gradient (1.077 g/mL) (Sigma) centrifugation and washed in IMDM with penicillin (100 U/mL), streptomycin (100 mg/mL) and amphotericin B (0.25 mg/mL) (Gibco Laboratories, Grand Island, NY USA) before and after treatment with RBC lysis buffer (155 mM NH4Cl, 10 mM NaHCO3, 0.1 mM EDTA). Mononuclear cells were resuspended in DMEM (Gibco) with 10% FBS and counted using capillary pipettes (BD Unopette).

### Mixed Lymphocyte Reaction

MSC, MSC-E, MSC-US2, MSC-US3, MSC-US6 and MSC-US11 were used as stimulator cells and PBMNC from human or sheep blood were used as responder cells. Prior to the experiment, each stimulator cell line was counted and its cell viability was assessed by trypan blue dye exclusion. 10^4^ cells of each stimulator cell line were plated in triplicate into a 96-well flat-bottom plate (BD Falcon) containing MSCGM™ and incubated until confluency was reached. They were then treated with 5 µg/ml mitomycin C (Roche Applied Science) at 37°C for 2.5 hrs in humidified incubator with 5% CO_2_ to prevent further proliferation. After incubation, cells were washed three times with IMDM. A co-culture was then made in triplicate by adding 1×10^5^ responder cells to each well, with a final volume of 0.1 ml of 10% FBS DMEM. The final MSC/PBMNC ratio per well used in this assay was approximately 1×10^4^/1×10^5^ = 1/10 in accordance with the recommendations of the BrdU cell proliferation colorimetric ELISA (Roche) technical bulletin.

Several controls were performed: media control and stimulator and responder controls, which contain either stimulator or responder cells alone. Cultures were incubated for 5 days at 37°C in 5% CO_2_ and 100% humidity. On the fifth day, BrdU (Roche, Mannheim, Germany) was added to each well to a final concentration of 10 µM. After incubation for 6 to 12 hrs at 37 C in 5% CO_2_ and 100% humidity, DNA synthesis was assayed with the BrdU cell proliferation colorimetric ELISA, (Roche) according to the manufacturer's instructions. A background control, which consists of incubating unlabeled stimulator cells with antibody against BrdU, was also performed. Newly synthesized BrdU-DNA was quantitated using a microplate reader (Bio-Rad, Hercules, CA).

### NK cytotoxicity assay

MSC, MSC-E, MSC-US2, MSC-US3, MSC-US6, MSC-US11 (1×10^5^ cells/ml; 50 µL/well) in a flat-bottomed 96-well microplate (BD Falcon) were incubated in triplicate with different concentrations of NK-92 MI cells (20×10^5^ cells/ml; 10×10^5^ cells/ml; 5×10^5^ cells/ml; 1×10^5^ cells/ml; 50 µL/well) in Alpha MEM complete media without phenol red (Gibco). All cells were previously assessed for cell viability. The cytotoxicity tests were run at 20∶1, 10∶1, 5∶1 and 1∶1 E∶T ratios following the guidelines of the CytoTox 96® Non-Radioactive Cytotoxicity Assay (Promega). The plate was centrifuged and incubated at 37°C for 4 hrs. Target cell maximum release was obtained by incubating each cell population with 9% Triton solution for 45 min at 37°C. The plate was centrifuged again and 50 µL of each supernatant was transferred to another flat bottom 96 well microplate containing 50 µL of substrate mix. Absorbance at 490 nm due to lactate dehydrogenase release was evaluated using a microplate reader (BioRad). Several controls were performed: (1) effector and target spontaneous controls, which contained of either effector or target cells alone; and (2) culture media background control. Culture medium background absorbance was subtracted from all absorbance values of Experimental, Target Cell Spontaneous Release, and Target Maximum Release. The percentage of specific lysis was calculated for all the different NK-MSC ratios as: Percent specific lysis = [ (experimental release−target spontaneous release)/(target maximal release−target spontaneous release)]×100.

### Transplantation of human MSC into fetal sheep recipients

MSC-E, MSC-US6 and MSC-US11 were cultured as previously described. A total of 5.6×10^4^ cells from each of the different MSC populations were injected in 0.4 ml of a QBSF60 serum-free medium (Quality Biologicals, Gaithersbrug, MD) into the peritoneal cavity of 61 day-old pre-immune fetal sheep recipients (term of 145 days) by ultrasound-guided injection with a transabdominal ultrasound apparatus (ALOKA SSD-100) outfitted with a 5-MHz probe. Each transduced MSC cell line was transplanted into two fetuses. At 66 days after transplantation, the animals were euthanized, and their tissues collected. Liver tissues were then analyzed for the presence of human donor cell engraftment, with MSC-E transplanted animals serving as controls. All sheep received humane care, and all the procedures were performed according to protocols approved by the Institutional Animal Care and Use Committee at the University of Nevada Reno (Protocol number 00314).

### Quantitative real time PCR to assess human engraftment

To perform the calibration curve for absolute qPCR, human MSC and sheep MSC were trypsinized and counted with Trypan Blue reagent (Amresco Inc., Branded Products Group, Solon, OH). Human and sheep MSC were then combined in different ratios to create a standard curve of samples with the following human cell concentrations: 0.01%, 0.1%, 0.5%, 1%, 5%, 10% and 20%, with a total of 2×10^6^ cells per each ratio. DNA from each cellular dilution was extracted, as was DNA from pure human MSC and pure sheep MSC (to serve as controls), using DNeasy blood and tissue kit (Qiagen Valencia, CA) according to the manufacturer's instructions. To perform the standard curve for GAPDH amplification and to detect human GAPDH copy numbers in the recipient tissues, we performed a 30 µl reaction consisting of 4.5 µl of Power SYBR® Green PCR Master Mix (SABioscience, Frederick, MD, USA), 0.2 µM GAPDH primers and 0.5 µg of each DNA. DNA was amplified in a MicroAmp® Optical 96-Well Reaction Plate using an Applied Biosystems Prism 7500 Fast Real-Time PCR System. After denaturizing at 95°C for 10 min, DNA products were amplified with 40 cycles, each of which consisted of denaturation at 95°C for 15 s, followed by annealing-extension at 60°C for 1 min. Quantitative measurements of GAPDH DNA were performed by kinetic PCR using SYBR® Green as fluorescent dye and ROX as passive reference dye. The accumulating DNA products were monitored by the ABI7500 sequence detection system (Applied Biosystems), and data were stored continuously during the reaction. The results were validated based on the quality of dissociation curves and good amplification efficiency of the primer. DNA was extracted from the collected liver tissues and each sample was analyzed in six replicates along with non-template controls to monitor for contaminating DNA. For each target tissue, the relative human GAPDH gene copy number was normalized with human GAPDH copy number obtained with DNA extracted from the liver of animals that received MSC-E cells, and was then analyzed by the 2-ΔΔCT method [67]. Each Ct value, obtained from human GAPDH DNA amplification for each recipient animal, was used to extrapolate from the standard curve the absolute human cell concentration for that animal.

### Tissue fixation and cryopreservation

Collected liver tissues were immersed in ice-cold PBS, washed three times and then immersed in 4% paraformaldehyde in PBS for 1 hour at 4°C. Subsequently, they were incubated in increasing sucrose concentrations in PBS (5%, 10%, 15%, and 20%), incubating for 30 min at 4°C for each sucrose concentration. Finally, the tissues were incubated in 2 parts 20% sucrose in PBS, one part OCT compound (Ted Pella, Inc. Redding, CA) and for 1 hour at 4°C with rocking and were then frozen in a cryomold, using liquid nitrogen-cooled isopentane.

### Detection of human engraftment by immunofluorescence

Frozen liver tissues were sectioned at a thickness of 8 µm using a cryostat and frozen sections were placed on microscopy slides. In order to stain the slide with immunofluorescence antibodies, frozen sections were first washed with 1× PBS at 4°C for three times, for five minutes each time. Non-specific binding sites within the tissues were then blocked by incubating the slides with 10% NGS, 1% BSA in 1× PBS for 1 hour at 4°C. Slides were washed twice for 5 minutes each with 2% NGS in 1× PBS. Sections were then incubated overnight at 4°C with primary antibody diluted appropriately in a solution consisting of 2% NGS in 1× PBS. After incubation with primary antibody, slides were washed three times, for 5 minutes each, in order to remove non-specifically bound primary antibody. The washing solution contained 2% NGS, 0.05% Triton in 1× PBS, followed by a last wash with only 2% NGS in 1× PBS. An appropriate fluorescently-labeled secondary antibody (5 µg/ml) was then added to the slides diluted in 2% NGS in 1× PBS, and slides were incubated for 1 hour at 4°C. Finally, slides were washed twice with 2% NGS in 1× PBS three times, for five minutes each, followed by a last wash with 1× PBS at 4°C. A couple of drops of DAPI (Biogene Ltd, Cambs, United Kindom) were added for 15 minutes to counterstain the nuclei within the section, followed by a wash with 1× PBS. Slides were then coverslipped with Cytoseal 60 (Thermo Fisher Scientific (NYSE: TMO)). Primary antibodies used for this study were the following: for NPT-II staining, rabbit anti-Neomycin Phosphotransferase II (NPT-II) (CR1112RP-500 ul) (Fitzgerald Industries International, MA, USA); for albumin staining, monoclonal anti-human serum albumin (clone hsa-9) (A2672); for Hepar-I staining, mouse monoclonal antibody (clone: OCH1E5) (Dako), and for Alpha fetoprotein staining, mouse monoclonal anti-human alpha 1 Fetoprotein antibody (Abcam). Secondary antibodies were the following: Alexa Fluor® 594 goat anti-rabbit IgG (A-11012) (Gibco – Invitrogen, Carlsbad, CA) and Alexa Fluor® 488 goat anti-mouse IgG (A-11001) (Gibco – Invitrogen, Carlsbad, CA). Images from each slide were taken using a Fluoview 1000 confocal microscope with a 40× objective (Olympus, Tokyo, Japan). Images were taken as z-stacks with 8–10 slices per image and then projected as 2-D images. Images were then processed using Adobe Photoshop. Sections from each tissue were stained with secondary antibody alone as negative control staining in all experiments.

### Statistical analysis

Experiments were independently repeated at least three times. Results are presented as mean± SEM. Unpaired two-tailed student T test and one-way ANOVA were used to analyze the statistical significance of the results; p values<0.05 were considered to be statistically significant. The Pearson correlation coefficient (R^2^) and the one-tailed p-value for the correlation coefficient were calculated to determine the correlation and significance, between human PBMNC proliferation and HLA-I MFI levels on the different MSC populations. p values<0.05 were considered to be statistically significant.

## Supporting Information

Figure S1 **Expression of HCMV US proteins by MSC decreases PBMNC proliferation.** Each of the transduced and untransduced MSCs were used as stimulators and were co-cultured with sheep PBMNC responders. After five days, DNA synthesis was assayed with the BrdU cell proliferation colorimetric ELISA. Data represents mean ± SEM of four independent experiments. In each experiment the specific stimulator-responder co-culture was performed in triplicate (* indicates p<0.01 and were considered statistically significant compared to MSC-E levels.
(TIF)
