# Supplementary figures and images for: Modulation of Human Mesenchymal Stem Cell Immunogenicity through Forced Expression of Human Cytomegalovirus US Proteins
Source: PLoS One. 2012 May 30;7(5):e36163. doi: 10.1371/journal.pone.0036163 (PMC3364258; doi:10.1371/journal.pone.0036163)

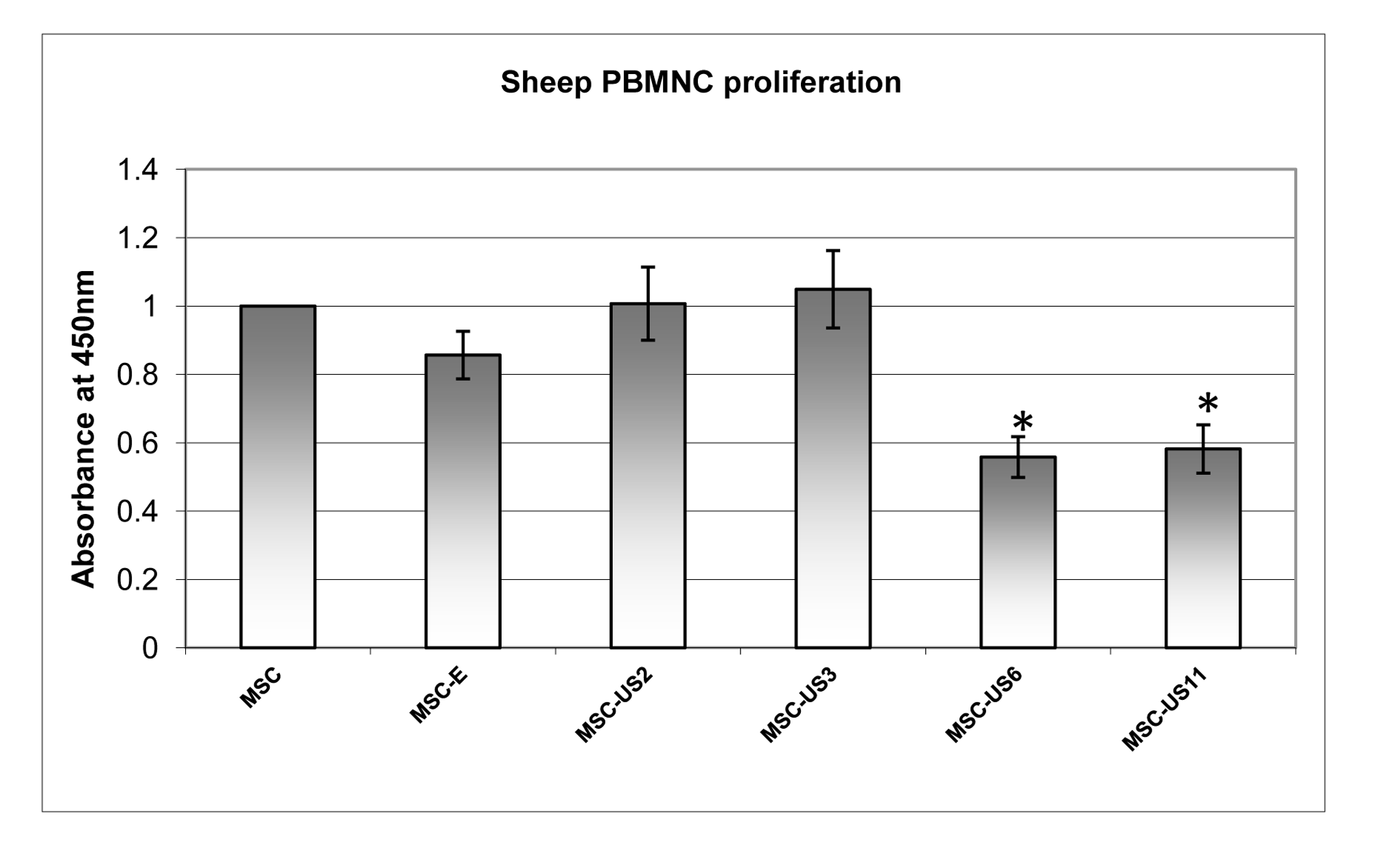

Supplement: Figure S1 — Expression of HCMV US proteins by MSC decreases PBMNC proliferation. Each of the transduced and untransduced MSCs were used as stimulators and were co-cultured with sheep PBMNC responders. After five days, DNA synthesis was assayed with the BrdU cell proliferation colorimetric ELISA. Data represents mean ± SEM of four independent experiments. In each experiment the specific stimulator-responder co-culture was performed in triplicate (* indicates p<0.01 and were considered statistically significant compared to MSC-E levels. (TIF) [file pone.0036163.s001.tif]
